# Something Attempted

**Published:** 1888-01-07

**Authors:** 


// The Hospital

A Weekly Institutional Journal of

Science, Medicine, and Philanthropy.

*For Hospitals, Asylums, Medical Practitioners, Students, Nurses, Families, Parishes, Congregations, and the Charitable Public.*

Vol. III. No. 67.] [January 7, 1888.

### "Something Attempted"—A Retrospect.

It is not always pleasant, but it is almost always profitable to go back upon past experience. We have now passed our second Christmas Day, and fully completed our first journalistic year. The period has been by no means uneventful, although Noachian deluges and other terrible convulsions of Nature have been mercifully spared us. Storms in tea-cups we have certainly been called upon to weather, and even billows of a more portentous magnitude have sometimes threatened to try our seamanship, but on the whole the voyage has been decidedly pleasant and more prosperous than either friends or enemies could by any possibility have anticipated. We hope we are not ungrateful.

The poor and the sick, their aids and their reliefs, their hospitals and their other charitable ameliorations have been our first care; a care, we consider, worthy of the best intelligence and the stoutest will which any man or woman is possessed of. In considering the "Empire of Benevolence," as it may be called, not only must its character and aims be taken into account, but also its vast extent and its almost infinite possibilities of wise and advantageous development. In Great Britain alone nearly five millions are annually expended in voluntary charity, a sum more than sufficient for the whole needs of large European states a few centuries ago. That this enormous sum is all wisely expended we do not say; but we do affirm that no other five millions which is annually expended can give a better account of itself. By far the larger portion of it is spent in work which is of a nature to be approved both by God and men.

Our work from week to week has vastly increased our knowledge of every class of benevolent institutions; and the increase of knowledge has corrected not a few erroneous impressions. So that the hospitals which, as a whole, we always felt to be worthy of public support, we now feel to be not only worthy of the utmost support, but also of unquestioning confidence. That is a bold word to say, but not more bold than true. Exceptions there doubtless are; but these are not merely few, they are also insignificant. The "cause of hospitals," to use a well-worn phrase, is one to stir the blood and awaken the purest, noblest, and most generous impulses of which men are capable. The work of hospitals calls into life some of the very highest and divinest forms of self-sacrificing duty. There are women at work within their walls, and men too, whose mere presence in the world brings sweetness and purity along with it. If busy and idle men and women would but now and again spare a little time to acquire some intelligent knowledge about hospitals, their workers and their work, they could not but be both instructed and thankful to know that such workers and such work are in their midst, and are the all-productive fruit of what are sometimes the mere remnants and fragments of a too-abundant wealth.

Although it has been our first care to make hospitals live in the eyes of the world, it has not been our only care. It is quite certain that those great, but partially understood, institutions have much to give as well as to receive; as much, or indeed more, than they receive. They give us highly-instructed medical men; but they give more; they give facts and principles of knowledge and experience which are worth more than gold to the world. Those facts and principles it has been our endeavour from week to week to expound in plain and simple language, so that whosoever would might easily make them his own. Not, of course, have we done this with the foolish and utterly impracticable idea of teaching every man to be his own doctor. We are not quite so ignorant of the elementary principles of civilisation, the distribution of labour, and the specialisation of function in the body-politic as that. One of our aims has been to throw such light on the general principles of medical practice, as pursued in hospitals, as should enable our non-professional readers to make the earliest and utmost use of the medical skill of the times. The doctor who has to deal with enlightened patients and families is not only saved a vast amount of unnecessary labour and anxiety, but is thereby assured of a very high average of success in his labours; whilst he whose work lies entirely among those who have no knowledge whatever of the general principles of medical treatment feels that though he has two eyes and a conscience he is almost as if he had not even one hand. "A little knowledge" may be "a dangerous thing;" but no knowledge at all is often fatal. Take, for example, the present epidemic of scarlet fever. Has it spread among the upper and middle classes who possess a certain understanding of medical treatment, and exercise a reasonable degree of care? Is it not well known that its extension has been almost entirely among the poor, and solely because they know little and care less even about the merest elements of medicine and sanitation? We conceive, then, that there is urgent public need for instruction in those general principles of medicine and sanitation which tend not only to conserve the public health, but also to enable the medical practitioner to get his instructions so intelligently and thoroughly carried out as to ensure the highest possible results of treatment in every case. From the commencement of our journalistic existence this has been one of our aims, as in the future it will still continue to be.

The Hospital circulates largely among medical practitioners, and it has appeared to us to be a duty to furnish them with certain "germs for thought," gathered from a wide area of literature or brought out from the stores of experience. Medicine is eminently an art which requires and lends itself to originality and individuality in its practice. The man of mere books and habits is usually a very inefficient practitioner. We admit that the man of no books is generally worse still. The most skilful is he who uses books as servants, and not as masters, as furnishing food for reflection and assimilation rather than as supplying fetters to bind him to routine. This is an age of fearless inquiry, bold speculation, and great courage in the initiation of new methods of practice. It is emphatically desirable that the mind of the whole profession be brought into critical relation with what is thought and done. "In the multitude of counsellors there is safety," and the adventurous few are more likely to render good service to their order and to the world when they are made to feel their full responsibility to the more sober and critical many. We desire to bring the average practitioner into actual and living relation with hospitals, and with what is done and taught at their bedsides from month to month. In so doing we have no intention of fostering intellectual idleness, or of lending ourselves to the practice of supplying what, in student language, are known as "tips." "Tips" of all kinds are abominations, which no man of sense will condescend to rely upon.

Much of what has been attempted for practitioners has also we have reason to believe been of service to students. The exposition and illustration of a general principle, or the singling out for special prominence and amplification of a disease or a symptom or a line of treatment will often illuminate whole regions which have hitherto appeared to the learner chaotic and dark. In our professional and clinical notes there has always been the intention of giving definite and practical aid to those who wish to follow their profession in a simple, straightforward, and modestly scientific spirit. True science, we conceive, does not necessarily confine itself to eminent persons, or live solely in learned phraseology. Simple facts, clearly understood and thoroughly believed in, with reasonable hypotheses and practical methods founded thereupon, are available for every honest and open-minded man's use. Believing this, we have striven to bring before our student readers facts and theories which seemed tous—the former verifiable, the latter sound.

As all our readers know, the *nursing* of patients, both in and out of hospital, has occupied no small share of the space of the journal, and the thought of its conductors. Nursing is not a separate art, but an integral and highly important part of medical practice. Whilst there has been much said on the subject which has been primarily of interest to nurses, there has always been the hope on the part of the writers that medical men, too, would read and profit. It is as necessary for a doctor to understand the theory and practice of nursing, as the theory and practice of drug prescribing. One of the special claims this journal has on the medical and student worlds is that it includes professional nursing in its teaching. All these departments of practical journalistic activity cover no small area, and require for their thorough working the best qualities of mind and heart. That they have been as thoroughly and intelligently worked as they ought to have been and might have been we should be the last to affirm; but that an honest attempt to do all which was possible with the means at disposal has been made none of our readers need for a moment doubt.

